# Cardiopulmonary Exercise Testing

**DOI:** 10.1155/2013/686104

**Published:** 2013-06-09

**Authors:** Darcy D. Marciniuk, Bruce D. Johnson, J. Alberto Neder, Denis E. O'Donnell

**Affiliations:** ^1^Division of Respirology, Critical Care and Sleep Medicine, University of Saskatchewan, Royal University Hospital, Saskatoon, SK, Canada S7N 0W8; ^2^Cardiovascular Diseases, Human Integrative and Environmental Physiology Laboratory, Mayo Clinic, Rochester, MN 55905, USA; ^3^Division of Respiratory & Critical Care Medicine, Queen's University, 102 Stuart Street, Kingston, ON, Canada K7L 2V6

Cardiopulmonary exercise testing (CPET) allows the clinician to objectively evaluate symptoms and important functions. CPET also arms the investigator with a powerful tool to better understand the respiratory system role as an engaged participant of a fully integrated physiologic system in humans. The insights gained mean that CPET can aid in the assessment of activity limitation and dyspnea, in evaluating disability and disease severity, in judging risk and prognosis, and in considering the suitability for specific interventions such as transplantation. In the research laboratory, CPET can contribute to the complete evaluation of new therapeutic or management agents, provide physiologic understanding for various hypotheses and new-found research results, and aid in discovery to further our understanding of the complex behaviour and interaction of the human respiratory system. While these benefits are well known to those working closely in the field, the need for greater and more effective dissemination of these benefits is required. Hence, this special issue focuses on cardiopulmonary exercise testing. 

We begin with M. K. Stickland et al. reviewing normal exercise physiology, as well as providing guidelines for the understanding of cardiopulmonary exercise test results. Techniques to ensure valid measurements of inspiratory capacity permitting a learned assessment of ventilatory constraint are outlined by J. A. Guenette and colleagues, while R. Ramos and others review the clinical usefulness of rapid incremental testing protocols. J. C. da Silva et al. examine the best criteria to determine ramp exercise testing speed in their original research study. Meanwhile, I. Vogiatzis and colleagues undertake a detailed review of varied mechanisms contributing to activity limitation in chronic lung diseases. B. Borel et al. explore the responsiveness of different exercise testing protocols to therapeutic interventions in COPD. A. Apostolo et al. review the interaction and consequences for the lungs in the setting of chronic heart failure, while C. H. Kim and colleagues report original research assessing the validity of a new multivariate index for grading gas exchange severity in patients with pulmonary arterial hypertension and with heart failure. Finally, D. E. O'Donnell et al. review the respiratory consequences of obesity and its impact on exercise performance in both health and in COPD.

We hope you find these articles both interesting and useful. We are confident that they will become meaningful resources in this field. 

